# Aspirin Eugenol Ester Ameliorates Fatty Liver Hemorrhagic Syndrome in Laying Hens by Reducing Oxidative Stress and Inflammation

**DOI:** 10.3390/ijms27114811

**Published:** 2026-05-27

**Authors:** Wenbo Ge, Kai Yan, Yajun Yang, Xiwang Liu, Xiao Xu, Shihong Li, Lixia Bai, Zhe Qin, Zhun Li, Di Lu, Jianyong Li

**Affiliations:** Key Laboratory of New Animal Drug of Gansu Province, Key Laboratory of Veterinary Pharmaceutical Development of Ministry of Agriculture and Rural Affairs, Lanzhou Institute of Husbandry and Pharmaceutical Sciences of Chinese Academy of Agricultural Sciences, Lanzhou 730050, China; gewenbo@caas.cn (W.G.); jinngoi17@gmail.com (K.Y.); yanyue10224@163.com (Y.Y.); xiwangliu@126.com (X.L.); xuxiao@caas.cn (X.X.); lzlishihong@163.com (S.L.); bailx552369@163.com (L.B.); qinzhe@caas.cn (Z.Q.); zhun2828@163.com (Z.L.); ludiludilu@163.com (D.L.)

**Keywords:** aspirin eugenol ester, fatty liver hemorrhagic syndrome, oxidative stress, inflammation, laying hen

## Abstract

Fatty liver hemorrhagic syndrome (FLHS) is a common metabolic disorder in laying hens, leading to reduced egg production and economic losses. Aspirin eugenol ester (AEE) has lipid-lowering, anti-inflammatory, and antioxidant properties, but its effects on FLHS are unknown. This study evaluated the protective effects of AEE using an in vivo FLHS model induced by a high-energy low-protein diet in laying hens and an in vitro steatosis model established by free fatty acid treatment in LMH cells. AEE alleviated liver histopathological damage, reduced oxidative stress (decreased ROS and MDA; increased SOD, GSH, and CAT), and suppressed inflammatory responses. The hepatoprotective effects of AEE were tentatively associated with altered molecular expression of the Nrf2 antioxidant pathway and MAPK/NF-κB inflammatory signaling; however, this correlation was speculated based on molecular detection and incomplete in vitro pharmacological interventions, lacking rigorous causal validation. These findings suggest that AEE alleviates FLHS-related liver injury in laying hens, possibly in association with altered oxidative and inflammatory status. Collectively, these preliminary findings provide a limited theoretical reference for the potential application of AEE as a preventive agent against FLHS in laying hens.

## 1. Introduction

Fatty liver hemorrhagic syndrome (FLHS) is a common nutritional and metabolic disease in laying hens, characterized by excessive hepatic lipid deposition and hemorrhage, which seriously impairs laying performance and causes substantial economic losses to the poultry industry [1,2]. Nutritional disorder is a major predisposing factor for FLHS; however, the exact pathogenesis of FLHS remains to be fully elucidated, limiting the development of targeted prevention strategies [3,4,5].

Mounting evidence indicates that oxidative stress and inflammatory response are closely involved in the occurrence and progression of FLHS [6]. Excessive reactive oxygen species accumulation breaks the hepatic antioxidant balance, further triggering inflammatory cascades and aggravating liver tissue damage [7,8,9]. Therefore, regulating oxidative stress and inflammation is considered a promising intervention direction for FLHS [10].

**Figure 1:**
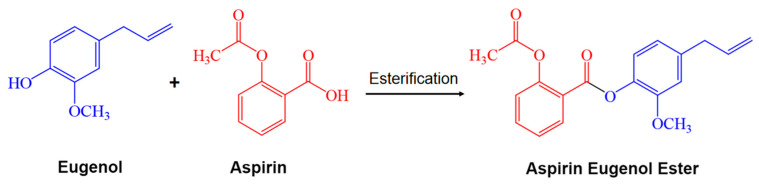
Synthesis of the aspirin eugenol ester.

AEE is a novel compound synthesized by esterifying aspirin with eugenol (Figure 1) [11]. AEE has demonstrated lipid-lowering, antioxidant, and anti-inflammatory activities [12,13]. Previous studies have confirmed the biological activities of AEE in rodent metabolic models and other poultry stress models [12,14]. Nevertheless, these existing findings cannot be directly extrapolated to FLHS, as FLHS has unique pathological characteristics distinct from rodent fatty liver and common broiler stress injury. To date, the protective effect and underlying mechanism of AEE against FLHS have remained unclear.

Given the above research gap, the present study was designed to explore the protective effect of AEE on FLHS and its regulatory role in oxidative stress and inflammation. Using an in vivo laying hen FLHS model and an in vitro hepatocyte steatosis model, this work aims to provide an experimental basis for the application and development of AEE in the prevention of FLHS.

## 2. Results

### 2.1. Pathological Changes of Liver

**Figure 2:**
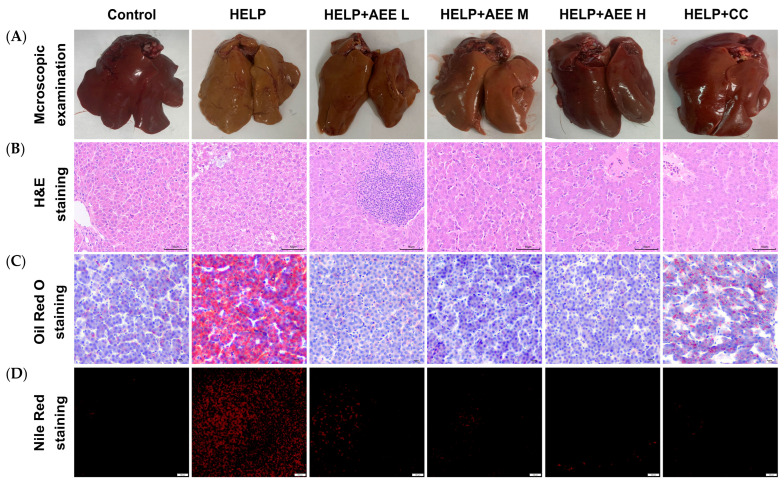
Pathological observation results of the liver: (**A**) Histopathological observation. (**B**) H&E staining (400×); yellow arrows mark lipid vacuoles. (**C**) Oil Red O staining (400×). (**D**) Nile Red staining (200×).

Compared with the control group, livers in the HELP group were enlarged, yellowish, and greasy and showed obvious hemorrhagic spots (Figure 2A). The HELP + CC group showed partial improvement, while AEE treatment, especially at the high dose, markedly reduced yellow discoloration and greasy texture, approaching the control group’s morphology. H&E staining revealed prominent hepatocellular vacuolation (lipid vacuoles, marked by yellow arrows) in the HELP group, which was attenuated in all AEE-treated groups (Figure 2B). The HELP + CC group also showed reduced vacuolation, though the improvement was less pronounced than in the HELP + AEE H group. Oil Red O and Nile Red staining showed minimal lipid droplets in the control group and extensive diffuse lipid accumulation in the HELP group. Lipid droplet deposition was diminished in all AEE groups, most notably in the HELP + AEE H group. Consistent across both stains, the HELP + AEE H group exhibited a greater reduction in lipid droplets than the HELP + CC group (Figure 2C,D). These findings suggested that the HELP diet induced obvious hepatic steatosis, while AEE alleviated such pathological alterations.

### 2.2. AEE Ameliorated HELP-Induced Hepatic Oxidative Stress and Inflammatory Response

**Figure 3:**
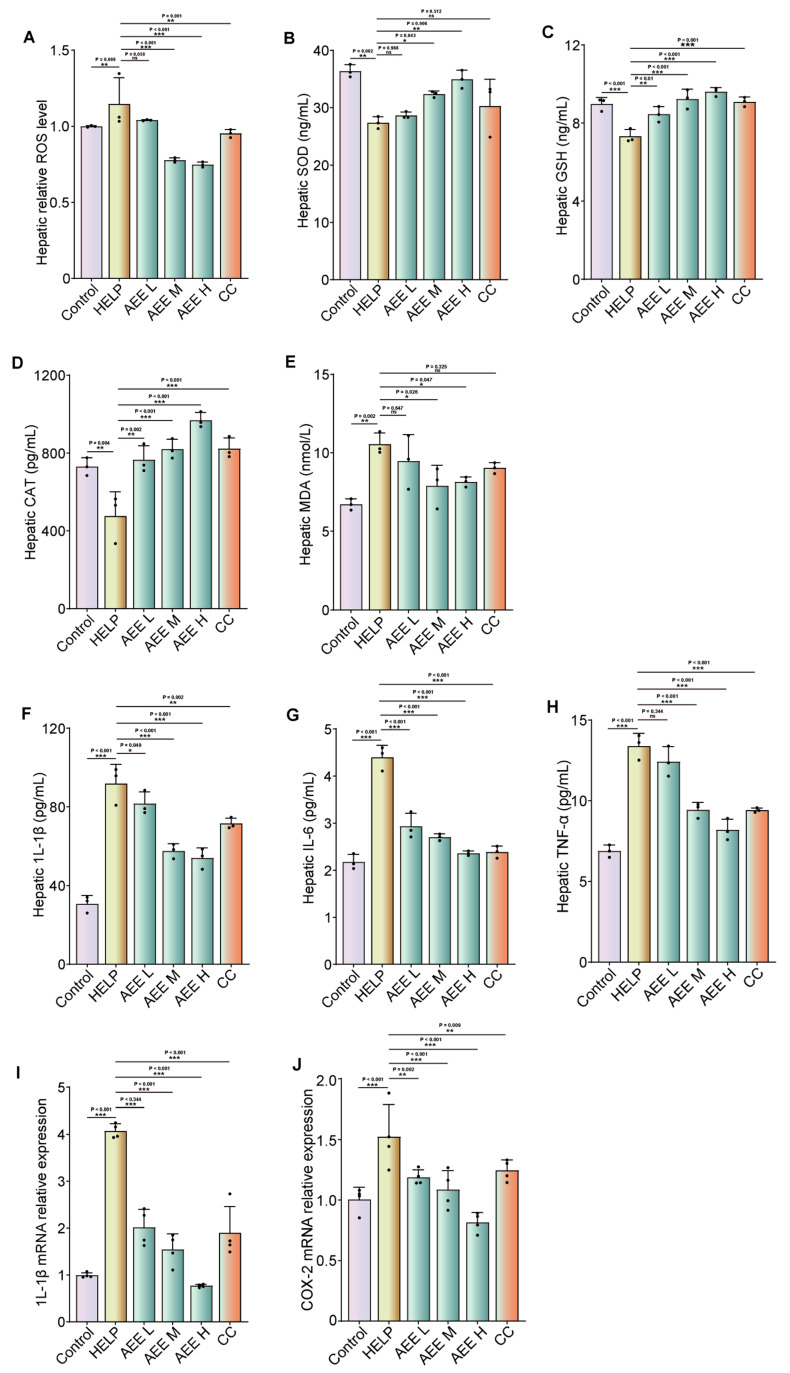
Effects of AEE on antioxidant capacity and inflammation: (**A**) Hepatic ROS levels. (**B**–**D**) Hepatic antioxidant enzyme activity. (**E**) Hepatic MDA content. (**F**) Hepatic IL-1β content. (**G**) Hepatic IL-6 content. (**H**) Hepatic TNF-α content. (**I**,**J**) The mRNA expression of IL-1β and COX-2. Data are presented as mean ± SEM. n = 3–4 per group, independent biological replicates. ns, not significant, * *p* < 0.05, ** *p* < 0.01, and *** *p* < 0.001.

To elucidate the involvement of oxidative stress and inflammation in the progression of FLHS in laying hens, we evaluated the corresponding biochemical and molecular indices. Compared with the control group, the HELP diet markedly elevated hepatic ROS levels, while AEE M, AEE H, and CC supplementation significantly suppressed this increase. The reduction in ROS levels was significantly more pronounced in the AEE M and AEE H groups than in the CC group (Figure 3A). The HELP diet significantly reduced the activities of antioxidant enzymes (SOD, GSH, and CAT) and increased the MDA level, indicating enhanced lipid peroxidation. These alterations were effectively reversed by AEE L, AEE M, AEE H, and CC treatment, with the increase in SOD activity being significantly more pronounced in the AEE M and AEE H groups than in the CC group (Figure 3B–E). Consistently, the HELP group exhibited significant upregulation of pro-inflammatory cytokines (IL-1β, IL-6, and TNF-α) and increased hepatic mRNA expression of IL-1β and COX-2, while AEE L, AEE M, AEE H, and CC supplementation markedly mitigated these inflammatory responses (Figure 3F–J). These findings demonstrate that AEE alleviates HELP-induced FLHS by suppressing oxidative stress and inflammatory signaling in the liver.

### 2.3. AEE Ameliorates FFA-Induced Oxidative Stress in LMH Cells and Its Possible Association with the Nrf2 Pathway

**Figure 4:**
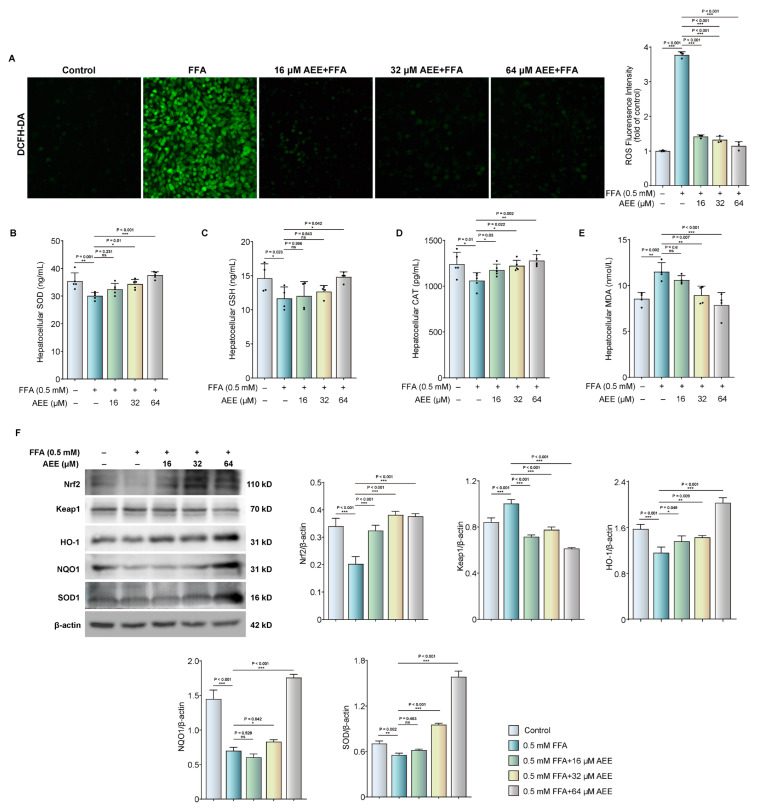
AEE alleviates FFA-induced oxidative stress in LMH cells with a potential association with the Nrf2 pathway: (**A**) ROS levels in LMH cells. (**B**–**D**) Antioxidant enzyme activities in LMH cells. (**E**) MDA content in LMH cells. (**F**) The protein expression levels of Nrf2, Keap1, HO-1, NQO1, and SOD1 were detected by Western blot. Data are presented as mean ± SEM. n = 3–5 per group, independent biological replicates. ns, not significant, * *p* < 0.05, ** *p* < 0.01, and *** *p* < 0.001.

To evaluate whether AEE mitigates FFA-induced oxidative stress in LMH cells, intracellular ROS levels were initially detected. As shown in Figure 4A, FFA exposure markedly increased ROS fluorescence intensity, whereas AEE (16, 32, and 64 μM) dose-dependently reduced this elevation. Consistent with these findings, FFA exposure significantly decreased the activities of antioxidant enzymes (SOD, GSH, and CAT) and increased MDA content. These alterations were partially reversed by AEE treatment (Figure 4B–E). At the molecular level, Western blot analysis revealed that AEE upregulated the protein expression of Nrf2, HO-1, NQO1, and SOD1, while downregulating Keap1 in FFA-stimulated LMH cells (Figure 4F). Furthermore, the Nrf2-specific inhibitor ML385 partially attenuated the above molecular changes caused by AEE (Supplementary Figure S1). Notably, this pharmacological intervention assay lacked independent inhibitor-only groups and matched vehicle controls; thus, non-specific interference and off-target effects of ML385 cannot be completely excluded. Collectively, these in vitro data preliminarily suggest a tentative association between Nrf2 signaling and the antioxidant capacity of AEE, without definitive evidence for explicit causal regulation.

### 2.4. AEE Alleviates FFA-Induced Inflammation in LMH Cells: Potential Association with MAPK and NF-κB Pathways

**Figure 5:**
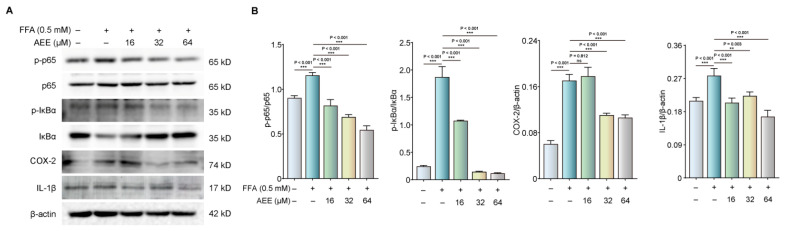
AEE alleviates FFA-induced inflammation in LMH cells with a potential association with the MAPK and NF-κB pathways: (**A**) The protein expression levels of p-p65, p65, p-IκB, IκB, COX-2, and IL-1β were detected by Western blot. (**B**) Data are presented as mean ± SEM. n = 3 per group, independent biological replicates. ns, not significant, ** *p* < 0.01 and *** *p* < 0.001.

To investigate the potential association between AEE and the MAPK/NF-κB inflammatory signaling, the activation status of these signaling cascades was determined. As shown in Figure 5A, FFA stimulation significantly increased the phosphorylation of IκBα and p65, as well as the expression of the pro-inflammatory mediators COX-2 and IL-1β. These alterations were partially reversed by AEE treatment in a dose-dependent manner. Meanwhile, FFA exposure markedly enhanced the phosphorylation of ERK, JNK, and p38, while AEE attenuated these phosphorylation events in a dose-dependent manner (Supplementary Figure S2A). At the molecular level, we further applied specific pathway inhibitors for auxiliary verification. Given the lack of independent inhibitor-only and vehicle control groups in this pharmacological intervention assay, non-specific effects of inhibitors cannot be completely ruled out. U0126 and SP600125 effectively suppressed FFA-induced activation of their respective kinases and significantly reduced NF-κB p65 phosphorylation, whereas SB203580 showed no similar effect (Supplementary Figure S2B–E). Collectively, these preliminary in vitro data suggest a potential association, rather than a definitive causal regulatory relationship, between MAPK/NF-κB signaling and the anti-inflammatory property of AEE.

### 2.5. AEE Alters Nrf2 and NF-κB Signaling Pathway in HELP-Fed Laying Hens

**Figure 6:**
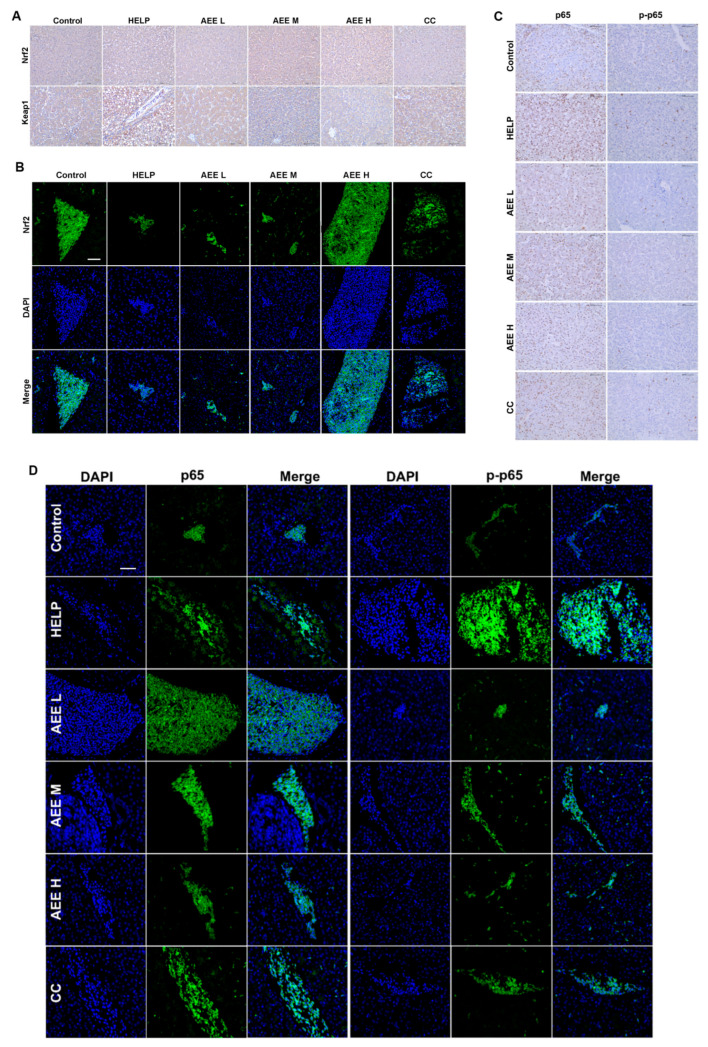
AEE affects the expression of Nrf2 and MAPK/NF-κB signaling-related molecules in HELP-fed laying hens: (**A**) Immunohistochemical staining of Nrf2 and Keap1 in the liver (400×). (**B**) Immunofluorescence staining of Nrf2 and Keap1 in the liver (200×). (**C**) Immunohistochemical staining of p-p65 and p65 in the liver (400×). (**D**) Immunofluorescence staining of p-p65 and p65 in the liver (200×).

To explore the in vivo effects of AEE on oxidative stress and inflammation-related molecular alterations, the distribution of core signaling proteins in hepatic tissues was detected. As shown in Figure 6A, the HELP diet induced obvious changes in hepatic Nrf2 and Keap1 expression levels. AEE treatment could partially reverse these alterations. Consistent with these findings, immunofluorescence staining further demonstrated that HELP feeding diminished Nrf2-related fluorescence signals, while AEE enhanced the signal intensity and distribution, particularly in the AEE H group (Figure 6B). For the NF-κB pathway, both immunohistochemical (Figure 6C) and immunofluorescent (Figure 6D) results showed that the HELP diet increased the expression and phosphorylation of p65, and such changes could be partially improved after AEE administration. The expression levels of key proteins involved in the Nrf2 and MAPK/NF-κB signaling pathways are presented in Supplementary Figure S3A–C. Consistent with the above in vitro results, these in vivo findings suggest a potential association, rather than a definite regulatory relationship, between AEE intervention and the varied activity of Nrf2 and NF-κB-related molecules in laying hens.

## 3. Discussion

FLHS is a common nutritional and metabolic disorder in high-yielding laying hens caused by nutritional imbalance and disrupted hepatic lipid homeostasis [15]. Although FLHS shares partial pathological characteristics with human NAFLD, including lipid accumulation, oxidative damage, and inflammatory activation, the interspecific differences limit the extrapolation of hen-derived experimental data to clinical liver research [16]. Nonetheless, exploring FLHS pathogenesis and effective protective agents is beneficial for the prevention of avian metabolic diseases and provides only limited preliminary references for NAFLD basic research.

In the present study, a HELP diet was successfully applied to establish a typical FLHS model accompanied by obvious hepatic steatosis and lipid deposition. Choline chloride was used as a positive control based on its well-documented hepatoprotective effects in poultry fatty liver injury [17]. Consistent with previous evidence [18,19,20,21], AEE intervention effectively ameliorated liver histological lesions and reduced excessive hepatic lipid accumulation. Notably, AEE exhibited better hepatoprotective efficacy than choline chloride under identical experimental conditions, indicating its promising application value for alleviating FLHS-associated liver damage.

Oxidative stress and impaired antioxidant defense are tightly involved in FLHS progression [22]. High-fat or imbalanced nutritional diets weaken hepatic antioxidant capacity and aggravate liver injury in both mice and laying hens [23,24,25]. Consistent with previous metabolic liver injury models, HELP and FFA stimulation induced redox imbalance, as evidenced by elevated ROS and MDA levels and decreased antioxidant enzyme activities. AEE dose-dependently restored dysregulated redox homeostasis in FLHS models. The Nrf2 pathway is a master regulator of cellular redox homeostasis [26]. Upon oxidative stress, Nrf2 dissociates from Keap1 and activates downstream antioxidant genes such as HO-1 and NQO1 via ARE binding [27]. In this study, AEE treatment was accompanied by altered expression profiles of Nrf2 and its downstream antioxidant molecules. Further cellular inhibition assays preliminarily indicated a potential association between Nrf2 signaling and the beneficial hepatic changes induced by AEE. Notably, the activation of Nrf2 is precisely controlled by nuclear translocation and ARE-dependent transcriptional activity [28]. Nevertheless, since only total protein levels were detected in the present study, detailed nucleocytoplasmic translocation and transcriptional activity of Nrf2 require further exploration.

Oxidative stress further triggers inflammatory responses and exacerbates liver damage [29,30]. Excess pro-inflammatory cytokines induced by the HELP diet were markedly decreased after AEE administration. The MAPK/NF-κB axis critically mediates inflammatory gene transcription, where activated ERK/JNK/p38 promotes IκBα and p65 phosphorylation [31,32]. AEE reduced the phosphorylation of MAPK and NF-κB-related molecules. Combined with inhibitor intervention results, the anti-inflammatory potential of AEE was tentatively correlated with expression variations within the ERK/JNK-NF-κB cascade. Notably, incomplete inhibitor control groups and limited biological replicates made it impossible to exclude non-specific drug interference. All pathway alterations observed in this study should be regarded as preliminary correlative changes rather than definitive causal regulations. The above observations are consistent with previous reports on natural compounds that modify oxidative and inflammatory signatures to ameliorate metabolic liver injury [33].

Limitations: This study has several inherent deficiencies. First, the LMH cell line and laying hen FLHS model cannot fully simulate long-term metabolic progression or complex systemic metabolism, restricting data extrapolation. Second, the absence of complete inhibitor controls and in vivo gene knockdown/knockout validation weakens the certainty of causal inferences regarding the involved pathways. Third, this study merely concentrated on two signaling cascades, while other underlying molecular mechanisms were not systematically investigated. Additionally, the dosage of choline chloride lacked gradient optimization, and pharmacokinetic information regarding AEE remains insufficient. Future studies will optimize experimental designs, expand detection indicators, and conduct field trials to further verify the practical application value of AEE.

## 4. Materials and Methods

### 4.1. Reagents

Aspirin eugenol ester (AEE) was synthesized by the Lanzhou Institute of Husbandry and Pharmaceutical Sciences of CAAS [11]. Choline chloride was purchased from Beijing Zhongtai Hongfeng Technology Co., Ltd. (Beijing, China). LMH cells were purchased from Shanghai Zhong Qiao Xin Zhou Biotechnology Co., Ltd. (Shanghai, China). 0.05% Trypsin-EDTA (25200072) and DMEM (6125298) basal medium were purchased from Gibco (Grand Island, NE, USA). Fetal Bovine Serum (AUS-01E-02) was purchased from Cell-Box (Changsha, China). BCA Protein Assay Kit (PC0020), Trizol^®^ (R1100), RIPA buffer (high) (R0010), SDS-PAGE loading buffer, 5× (with DTT) (P1040), and 100 IU/mL penicillin and 100 μg/mL streptomycin were purchased from Solarbio (Beijing, China). PrimeScript™ RT Master Mix (RR036A) and TB Green^®^ Premix Ex Taq™ II FAST qPCR (CN830A) were purchased from Takara (Nanjing, China). Oleic acid (O1008-25g) and palmitic acid (8005081000) were purchased from Sigma-Aldrich (St. Louis, MO, USA). ML385 (HY-100523), SP600125 (HY-12041), SB203580 (HY-10256), U0126 (HY-12031A), and PDTC (HY-18738) were purchased from Med Chem Express (Shanghai, China). PVDF membrane (ISEQ00010) was purchased from Merck-Millipore (Burlington, MA, USA). DAPI (C1002) was purchased from Beyotime (Nanjing, China). Detailed information on all antibodies used in this study is listed in Supplementary Table S1.

### 4.2. Animals and Experimental Design

A total of seventy-two healthy 170-day-old Hy-Line Brown laying hens were obtained from a commercial farm. Following a one-week acclimatization period under controlled environmental conditions (temperature 20–22°C; 16 h light/8 h dark photoperiod), the hens were randomly assigned to six dietary groups using a random number table. Each group contained four independent replicate pens with three hens per pen. Each individual hen was defined as a biologically independent experimental unit for statistical analysis. Hens within the same pen were physically separated and managed individually to eliminate pen clustering effects.

The six groups were as follows: (1) control group: fed a basal diet; (2) HELP group: fed a high-energy low-protein (HELP) diet to induce FLHS [34]; (3–5) AEE-treated group: fed the HELP diet supplemented with AEE at 25 (AEE L), 50 (AEE M), or 100 (AEE H) mg/kg body weight, respectively; (6) choline chloride (CC) group: fed the HELP diet supplemented with CC at 2 g/kg of diet. Previous studies have confirmed that the optimal dose of AEE in rats is 54 mg/kg [13], which was converted to laying hens using the body surface area method, yielding a reference dose of 50 mg/kg. Therefore, the doses of AEE used in this study were 25, 50, and 100 mg/kg (low, medium, and high doses, respectively). The basal diet was formulated according to the recommendations of the National Research Council [35]. The FLHS model was induced using a high-energy, low-protein (HELP) diet based on a previously described formulation [34], with slight modifications. The compositions of the basal and HELP diets are provided in Table 1. The experiment lasted for 90 days. The hens were fed once daily, and water was available ad libitum. Blinded analysis was performed to avoid subjective judgment.

### 4.3. Sample Collection

At the end of the 90-day experimental period, laying hens were fasted for 12 h and subsequently euthanized. The entire liver from each hen was photographed for morphological documentation. Liver samples were collected and either fixed in 4% paraformaldehyde for histological examination or rapidly frozen in liquid nitrogen and stored at −80 °C until further analysis.

### 4.4. Cell Culture and Treatments

LMH cells were maintained in DMEM supplemented with 10% fetal bovine serum, 100 U/mL penicillin, and 100 μg/mL streptomycin. Cultures were incubated at 37°C in a humidified atmosphere containing 5% CO_2_. AEE was dissolved in dimethyl sulfoxide (DMSO) at a final concentration of ≤0.1% (*v*/*v*) in culture medium. The control group received an equivalent volume of DMSO (0.1% *v*/*v*) as solvent control to exclude any potential solvent effects.

To induce cellular steatosis, cells were exposed to 0.5 mM free fatty acids (FFAs) for 24 h. The FFAs were prepared as a mixture of oleic acid and palmitic acid (2:1, molar ratio) complexed with fatty acid-free BSA.

To assess the protective effects of AEE, cells were pretreated with 16, 32, and 64 μM AEE for 24 h prior to FFA exposure. These concentrations were chosen based on CCK-8 cytotoxicity results to ensure non-toxic working ranges. For mechanism studies, cells were preincubated for 1 h with specific pharmacological modulators or their respective vehicle controls before subsequent treatments. The following modulators were used: 5 μM ML385 (Nrf2 inhibitor), 20 μM SP600125 (JNK inhibitor), 20 μM SB203580 (p38 inhibitor), 10 μM U0126 (ERK1/2 inhibitor), and 50 μM PDTC (NF-κB inhibitor). Although inhibitor-only and vehicle control groups were not included in the current experimental design, the specificity and low cytotoxicity of these inhibitors at the concentrations used have been extensively validated in previous reports [32,36]. This limitation is acknowledged in the discussion.

### 4.5. Oxidative Stress Level Evaluation

Hepatic and cellular oxidative stress indicators were quantified using commercial assay kits. The levels of malondialdehyde (MDA), catalase (CAT), superoxide dismutase (SOD), and glutathione (GSH) in liver tissues and cells were measured with enzyme-linked immunosorbent assay (ELISA) kits (Mlbio Biotechnology Co., Ltd., Shanghai, China). Reactive oxygen species (ROS) levels in liver tissues were measured using a specific assay kit (Bestbio Co., Ltd., Shanghai, China). All procedures were strictly performed in accordance with the manufacturers’ protocols.

### 4.6. Inflammatory Cytokine Level Evaluation

The concentrations of key proinflammatory cytokines, including interleukin-1β (IL-1β), interleukin-6 (IL-6), and tumor necrosis factor-α (TNF-α), in liver tissues were detected using ELISA kits (Jianglai Biotechnology Co., Ltd., Shanghai, China), following the manufacturers’ protocols.

### 4.7. Histological Examination

For histological evaluation, liver tissues were fixed in 4% paraformaldehyde for 24 h and subsequently embedded in paraffin. Sections of 5 μm thickness were prepared and stained with hematoxylin and eosin (H&E) as previously described [37]. The stained sections were imaged under a light microscope.

### 4.8. Oil Red O and Nile Red Staining

To assess hepatic steatosis, Oil Red O staining was performed on frozen liver sections, as previously described [38]. Briefly, frozen liver sections were equilibrated to room temperature for 10 min and rinsed with distilled water. Cultured cells were washed twice with PBS, fixed with 4% paraformaldehyde for 20 min, and then rinsed twice with distilled water. Both liver and cell samples were immersed in 60% isopropanol for 30 s, followed by staining with an Oil Red O working solution for 20 min. Excess dye was removed with 60% isopropanol. Nuclei were counterstained with hematoxylin for 2 min and washed under running tap water for 10 min to develop the blue color. Finally, samples were rinsed with distilled water, mounted with glycerin gelatin, and photographed using an optical microscope. Nile Red staining was performed according to a previous report [39].

### 4.9. Immunohistochemistry

Liver tissue samples were fixed, dehydrated, cleared, and embedded in paraffin according to standard histological procedures. Paraffin sections (5 μm) were deparaffinized, rehydrated through a graded ethanol series, and subjected to antigen retrieval. Endogenous peroxidase activity was blocked with 3% hydrogen peroxide, followed by incubation with normal serum for 30 min at room temperature to reduce nonspecific binding. The sections were then incubated overnight at 4 °C with primary antibodies against Nrf2, Keap1, p65, and p-p65 (1:150 dilution). After washing, the sections were incubated with horseradish peroxidase (HRP)-conjugated secondary antibodies for 30 min at room temperature. Immunoreactive signals were visualized using a DAB substrate kit, and nuclei were counterstained with hematoxylin. Finally, the sections were dehydrated, cleared, mounted, and examined under a light microscope.

### 4.10. Immunofluorescence Staining

Liver tissue sections were deparaffinized and treated with 3% H_2_O_2_ to inactivate endogenous peroxidase activity. Both tissue sections and cultured cells were blocked with 5% BSA for 1 h at room temperature. Prior to blocking, cells were washed three times with PBS and fixed with 4% paraformaldehyde for 20 min. After washing with PBS, all samples were permeabilized with 0.3% Triton X-100 for 15 min and then incubated overnight at 4 °C with primary antibodies against p-p65, p65, and Nrf2 (1:100 dilution). Following incubation, the samples were washed and incubated with Alexa Fluor 488- or Alexa Fluor 594-conjugated goat anti-rabbit/mouse IgG secondary antibodies at 37 °C for 45 min. Nuclei were counterstained with DAPI, and images were captured using a fluorescence microscope.

### 4.11. Quantitative Real-Time PCR

Quantitative Real-Time PCR (qRT-PCR) was performed as described previously [40]. Briefly, total RNA was extracted from liver tissues using TRIzol reagent. cDNA was synthesized using the PrimeScript RT Reagent Kit following the manufacturer’s instructions. qPCR was carried out using SYBR Green PCR Master Mix on an ABI 7500 Real-Time PCR system (Applied Biosystems, Carlsbad, CA, USA). Primer sequences are listed in Table 2. The relative mRNA expression levels of target genes were normalized to *β-actin* as an internal control and calculated using the 2^−ΔΔCt^ method [41]. All the results were obtained from at least three independent experiments.

### 4.12. Western Blotting

Total proteins from cells and liver tissues were extracted using standard protocols. Protein concentrations were determined with a BCA Protein Assay Kit. Equal amounts of protein were separated by SDS-PAGE and transferred onto PVDF membranes. After blocking, the membranes were incubated overnight at 4 °C with the primary antibodies. Subsequently, the membranes were incubated with horseradish peroxidase (HRP)-conjugated secondary antibodies (1:3000; Proteintech, Wuhan, China) for 1 h at room temperature. Immunoreactive bands were visualized using an enhanced chemiluminescence (ECL) detection system and imaged with an Amersham Imager 600 (GE Healthcare Bio-Sciences, Uppsala, Sweden). Band intensities were quantified using ImageJ software 1.38.

### 4.13. Measurement of Intracellular ROS

Intracellular ROS levels were determined using a ROS assay kit (S0033, Beyotime, Nanjing, China) according to the manufacturer’s instructions. This method is based on the oxidation of dichlorodihydrofluorescein (DCFH) to dichlorofluorescein (DCF), which emits green fluorescence upon excitation at 488 nm. After the indicated treatments, fluorescence images were captured using a fluorescence microscope (Revolve Omega, Apexbio, Suzhou, China).

### 4.14. Statistical Analysis

Data are expressed as the mean ± SEM. All statistical analyses were performed using SPSS software (version 19.0; IBM, Armonk, NY, USA) and GraphPad Prism (version 9.0; San Diego, CA, USA). One-way analysis of variance (ANOVA) was performed, followed by Dunnett’s post hoc test for multiple comparisons. Statistical significance was defined as *p* < 0.05, and exact *p*-values as well as effect sizes were reported where appropriate. The number of independent biological replicates for each assay is explicitly stated in the corresponding figure legends. All experimental procedures, including sample collection, biochemical assays, and histological and molecular analyses, were performed by investigators blinded to the group allocation.

## 5. Conclusions

In summary, this study suggests that oxidative stress and inflammation are closely involved in the pathogenesis of FLHS in laying hens. AEE ameliorates FLHS-related pathological damage under experimental conditions. These beneficial effects are preliminarily associated with altered expression of Nrf2-related antioxidant and MAPK/NF-κB inflammatory molecules, rather than demonstrating definitive causal regulation. Collectively, these preliminary findings provide a limited theoretical reference for exploring the potential application of AEE in the prevention of FLHS in laying hens. Further rigorous mechanistic verification with improved experimental control designs, sufficient biological replicates, and additional molecular validation approaches is still required to clarify the precise molecular mechanisms.

## Figures and Tables

**Table 1:** Diet composition and nutrient levels (air-dry basis, %).

Item	Basic Diet	High-Energy Low-Protein Diet
Ingredients		
Corn	64.00	70.00
Soybean meal	26.00	15.78
Soybean oil	0.00	4.22
Calcium	8.00	8.00
Premix *	2.00	2.00
Total	100.00	100.00
Nutrient levels		
Crude protein	15.5	12.3
Available phosphorus	0.53	0.51
Arginine	1.03	0.74
Methionine	0.37	0.32
Valine	0.77	0.58
Metabolic energy (kcal/kg)	2687.30	3156.40
Met + Cys	0.67	0.56

* Per kilogram of additives contained the following: Cu, 2.50 mg; Fe, 20.00 mg; Zn, 17.50 mg; Mn, 15.00 mg; KI, 4.00 mg; Na_2_SeO_3_, 6.00 mg; CoCl_2_·6H_2_O, 2.5 mg; Met, 50.00 mg; chromium, 2.00 mg; phytase, 10.00 mg; kininase, 7.50 mg; antioxidant, 2.00 mg; betaine, 15.00 mg; choline, 50.00 mg; NaCl, 200.00 mg; Ca-P, 500.00 mg; zeolite, 76.00 mg.

**Table 2:** Primers used for qRT-PCR.

Gene	Sequence (5′-3′)	Accession Number
*IL-1β*	F: ACTGGGCATCAAGGGCTA	AJ245728
	R:GGTAGAAGATGAAGCGGGTC	
*COX-2*	F: TGTCCTTTCACTGCTTTCCAT	MN013407.1
	R:TTCCATTGCTGTGTTTGAGGT	
*β-actin*	F:CCGCTCTATGAAGGCTACGC	NM_205518.1
	R:CTCTCGGCTGTGGTGGTGAA	

## Data Availability

The datasets analyzed during the current study are available from the corresponding author upon reasonable request.

## Supplementary Materials

The following supporting information can be downloaded at: https://www.mdpi.com/article/10.3390/ijms27114811/s1.

## Abbreviations

The following abbreviations are used in this manuscript:

AEE	aspirin eugenol ester
FLHS	fatty liver hemorrhagic syndrome
HELP	high-energy low-protein
FFA	free fatty acid
MDA	malondialdehyde
CAT	catalase
SOD	superoxide dismutase
GSH	glutathione
ROS	reactive oxygen species
IL-1β	interleukin-1 beta
IL-6	interleukin-6
TNF-α	tumor necrosis factor-alpha
H&E	hematoxylin and eosin
CCK-8	Cell Counting Kit-8

## References

[1] Gao X., Liu S., Ding C., Miao Y., Gao Z., Li M., Fan W., Tang Z., Mhlambi N.H., Yan L. (2021). Comparative effects of genistein and bisphenol A on non-alcoholic fatty liver disease in laying hens. Environ. Pollut..

[2] Shini A., Shini S., Bryden W.L. (2019). Fatty liver haemorrhagic syndrome occurrence in laying hens: Impact of production System. Avian Pathol..

[3] Gretarsson P., Kittelsen K., Moe R.O., Vasdal G., Toftaker I. (2023). End of lay postmortem findings in aviary housed laying hens. Poult. Sci..

[4] Grimes T.M. (1975). Causes of disease in two commercial flocks of laying hens. Aust. Vet. J..

[5] Zhang S., Zhao X., He X., Shi W., Ma N. (2026). Metabolite-mediated crosstalk: Unraveling the interactions between gut microbiota and host in fatty liver hemorrhagic syndrome of laying hens. J. Anim. Sci. Biotechnol..

[6] Ding J., Liu J., Chen J., Cheng X., Cao H., Guo X., Hu G., Zhuang Y. (2024). Sodium butyrate alleviates free fatty acid-induced steatosis in primary chicken hepatocytes via the AMPK/PPARα pathway. Poult. Sci..

[7] Cui Y., Ru M., Wang Y., Weng L., Haji R.A., Liang H., Zeng Q., Wei Q., Xie X., Yin C. (2024). Epigenetic regulation of H3K27me3 in laying hens with fatty liver hemorrhagic syndrome induced by high-energy and low-protein diets. BMC Genom..

[8] Scalise V., Sanguinetti C., Neri T., Cianchetti S., Lai M., Carnicelli V., Celi A., Pedrinelli R. (2021). PCSK9 induces tissue factor expression by activation of TLR4/NFkB signaling. Int. J. Mol. Sci..

[9] Zhou M., Qiang J., Gan J., Xu X., Li X., Zhang S., Xu B., Dong Z. (2023). Quercetin attenuates environmental Avermectin-induced ROS accumulation and alleviates gill damage in carp through activation of the Nrf2 pathway. Comp. Biochem. Physiol. C Toxicol. Pharmacol..

[10] Wang N., Li W., Ouyang G., Li H., Yang J., Wu G. (2025). Goose Deoxycholic Acid Ameliorates Liver Injury in Laying Hens with Fatty Liver Hemorrhage Syndrome by Inhibiting the Inflammatory Response. Int. J. Mol. Sci..

[11] Li J., Yu Y., Wang Q., Zhang J., Yang Y., Li B., Zhou X., Niu J., Wei X., Liu X. (2012). Synthesis of aspirin eugenol ester and its biological activity. Med. Chem. Res..

[12] Guo C., Zhang Y., Bai D., Zhen W., Ma P., Wang Z., Zhao X., Ma X., Xie X., Ito K. (2025). Aspirin Eugenol Ester Alleviates Energy Metabolism Disorders by Reducing Oxidative Damage and Inflammation in the Livers of Broilers Under High-Stocking-Density Stress. Int. J. Mol. Sci..

[13] Karam I., Ma N., Liu X., Kong X., Zhao X., Yang Y., Li J. (2016). Lowering effects of aspirin eugenol ester on blood lipids in rats with high fat diet. Lipids Health Dis..

[14] Zhang H., Zhang Y., Bai D., Zhong J., Hu X., Zhang R., Zhen W., Ito K., Zhang B., Yang Y. (2024). Effect of dietary aspirin eugenol ester on the growth performance, antioxidant capacity, intestinal inflammation, and cecal microbiota of broilers under high stocking density. Poult. Sci..

[15] Rozenboim I., Mahato J., Cohen N.A., Tirosh O. (2016). Low protein and high-energy diet: A possible natural cause of fatty liver hemorrhagic syndrome in caged White Leghorn laying hens. Poult. Sci..

[16] Hamid H., Zhang J., Li W., Liu C., Li M., Zhao L., Ji C., Ma Q. (2019). Interactions between the cecal microbiota and non-alcoholic steatohepatitis using laying hens as the model. Poult. Sci..

[17] Aziza A.E., Awadin W., Cherian G. (2019). Impact of Choline Supplementation on Hepatic Histopathology, Phospholipid Content, and Tocopherol Status in Layer Hens Fed Flaxseed. J. Appl. Poult. Res..

[18] Qiu K., Zhao Q., Wang J., Qi G., Wu S., Zhang H. (2021). Effects of Pyrroloquinoline Quinone on Lipid Metabolism and Anti-Oxidative Capacity in a High-Fat-Diet Metabolic Dysfunction-Associated Fatty Liver Disease Chick Model. Int. J. Mol. Sci..

[19] You M., Zhang S., Shen Y., Zhao X., Chen L., Liu J., Ma N. (2023). Quantitative lipidomics reveals lipid perturbation in the liver of fatty liver hemorrhagic syndrome in laying hens. Poult. Sci..

[20] Karam I., Ma N., Liu X., Li S., Kong X., Li J., Yang Y. (2015). Regulation effect of Aspirin Eugenol Ester on blood lipids in Wistar rats with hyperlipidemia. BMC Vet. Res..

[21] Lu X., Tao Q., Qin Z., Liu X., Li S., Bai L., Ge W., Liu Y., Li J., Yang Y. (2024). A combined transcriptomics and proteomics approach to reveal the mechanism of AEE relieving hyperlipidemia in ApoE^−/−^ mice. Biomed. Pharmacother..

[22] Shini S., Shini A., Bryden W.L. (2019). Unravelling Fatty Liver Haemorrhagic Syndrome: 2. Inflammation and Pathophysiology. Avian Pathol..

[23] Ye Q., Liu Y., Zhang G., Deng H., Wang X., Tuo L., Chen C., Pan X., Wu K., Fan J. (2023). Deficiency of gluconeogenic enzyme PCK1 promotes metabolic-associated fatty liver disease through PI3K/AKT/PDGF axis activation in male mice. Nat. Commun..

[24] Yuan Y., He J., Tang M., Chen H., Wei T., Zhang B., Liang D., Nie X. (2023). Preventive effect of Ya’an Tibetan tea on obesity in rats fed with a hypercaloric high-fat diet revealed by gut microbiology and metabolomics studies. Food Res. Int..

[25] Miao S., Mu T., Li R., Li Y., Zhao W., Li J., Dong X., Zou X. (2024). Coated sodium butyrate ameliorates high-energy and low-protein diet induced hepatic dysfunction via modulating mitochondrial dynamics, autophagy and apoptosis in laying hens. J. Anim. Sci. Biotechnol..

[26] Zhou J., Zheng Q., Chen Z. (2022). The Nrf2 pathway in liver diseases. Front. Cell Dev. Biol..

[27] Saha S., Buttari B., Panieri E., Profumo E., Saso L. (2020). An overview of Nrf2 signaling pathway and its role in inflammation. Molecules.

[28] Franceschetti L., Bonomini F., Rodella L.F., Rezzani R. (2020). Critical Role of NFκB in the Pathogenesis of Non-alcoholic Fatty Liver Disease: A Widespread Key Regulator. Curr. Mol. Med..

[29] Ge C., Xu M., Qin Y., Gu T., Feng J., Lv J., Wang S., Ma Y., Lou D., Li Q. (2019). Loss of RIP3 initiates annihilation of high-fat diet initialized nonalcoholic hepatosteatosis: A mechanism involving Toll-like receptor 4 and oxidative stress. Free Radic. Bio. Med..

[30] Li D., Meng K., Liu G., Wen Z., Han Y., Liu W., Xu X., Song L., Cai H., Yang P. (2025). *Lactiplantibacillus plantarum* FRT4 protects against fatty liver hemorrhage syndrome: Regulating gut microbiota and FoxO/TLR-4/NF-κB signaling pathway in laying hens. Microbiome.

[31] Zhao H., Tian H. (2024). Icariin alleviates high-fat diet-induced nonalcoholic fatty liver disease via up-regulating miR-206 to mediate NF-κB and MAPK pathways. J. Biochem. Mol. Toxicol..

[32] Qin S., Yang C., Huang W., Du S., Mai H., Xiao J., Lü T. (2018). Sulforaphane attenuates microglia-mediated neuronal necroptosis through down-regulation of MAPK/NF-κB signaling pathways in LPS-activated BV-2 microglia. Pharmacol. Res..

[33] Fang Z., Xu H., Duan J., Ruan B., Liu J., Song P., Ding J., Xu C., Li Z., Dou K. (2023). Short-term tamoxifen administration improves hepatic steatosis and glucose intolerance through JNK/MAPK in mice. Signal Transduct. Target. Ther..

[34] Xing Y., Huang B., Cui Z., Zhang Q., Ma H. (2024). Dioscin improves fatty liver hemorrhagic syndrome by promoting ERα-AMPK mediated mitophagy in laying hens. Phytomedicine.

[35] National Research Council (1969). Nutrient Requirements of Poultry: Ninth Revised Edition, 1994.

[36] Wei Y., Zhang Y., Zhan B., Wang Y., Cheng J., Yu H., Lv M., Zhang Y., Zhai Y., Guan Y. (2025). Asiaticoside alleviated NAFLD by activating Nrf2 and inhibiting the NF-κB pathway. Phytomedicine.

[37] Lai C., Ho M.H., Tsai M.L., Li S., Badmaev V., Ho C.T., Pan M.H. (2013). Suppression of adipogenesis and obesity in high-fat induced mouse model by hydroxylated polymethoxyflavones. J. Agric. Food Chem..

[38] Jin L., Fang W., Li B., Shi G., Li X., Yang Y., Yang J., Zhang Z., Ning G. (2012). Inhibitory effect of andrographolide in 3T3-L1 adipocytes differentiation through the PPARγ pathway. Mol. Cell. Endocrinol..

[39] Herms A., Bosch M., Reddy B.J.N., Schieber N.L., Fajardo A., Rupérez C., Fernández-Vidal A., Ferguson C., Rentero C., Tebar F. (2015). AMPK activation promotes lipid droplet dispersion on detyrosinated microtubules to increase mitochondrial fatty acid oxidation. Nat. Commun..

[40] Chang T., Chiang H., Lai Y., Huang Y., Huang H., Liang Y., Liu H., Huang C. (2019). *Helminthostachys zeylanica* alleviates hepatic steatosis and insulin resistance in diet-induced obese mice. BMC Complement. Altern. Med..

[41] Zhu Y., Zhang X., Du P., Wang Z., Luo P., Huang Y., Liu Z., Zhang H., Chen W. (2022). Dietary herbaceous mixture supplementation reduced hepatic lipid deposition and improved hepatic health status in post-peak laying hens. Poult. Sci..

