# Supplementary material for: Aspirin Eugenol Ester Ameliorates Fatty Liver Hemorrhagic Syndrome in Laying Hens by Reducing Oxidative Stress and Inflammation
Source: Int J Mol Sci. 2026 May 27;27(11):4811. doi: 10.3390/ijms27114811 (PMC13257362; doi:10.3390/ijms27114811)
Supplement: Supplementary file 1 [file ijms-27-04811-s001.zip › ijms-4298714-supplementary.pdf]

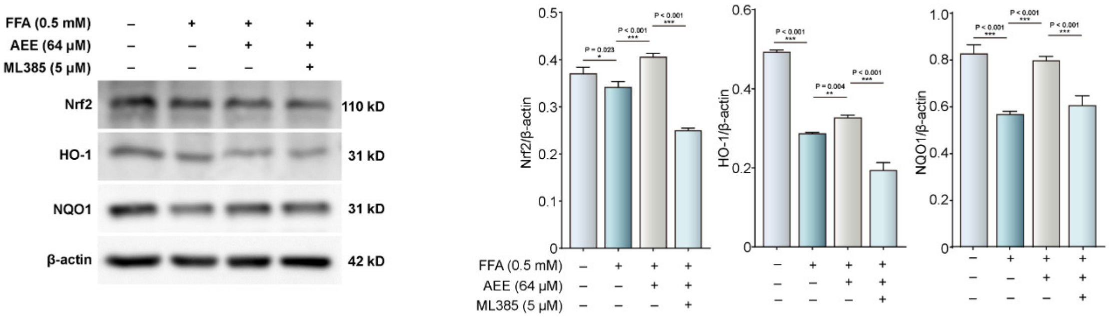

Supplementary Figure S1. Effects of ML385 on Nrf2 pathway-related proteins. The protein expression levels of Nrf2, HO-1, and NQO1 were detected by Western blot. Data are presented as mean ± SEM. n = 3 per group, independent biological replicates. * $P < 0.05$, ** $P < 0.01$, and *** $P < 0.001$.

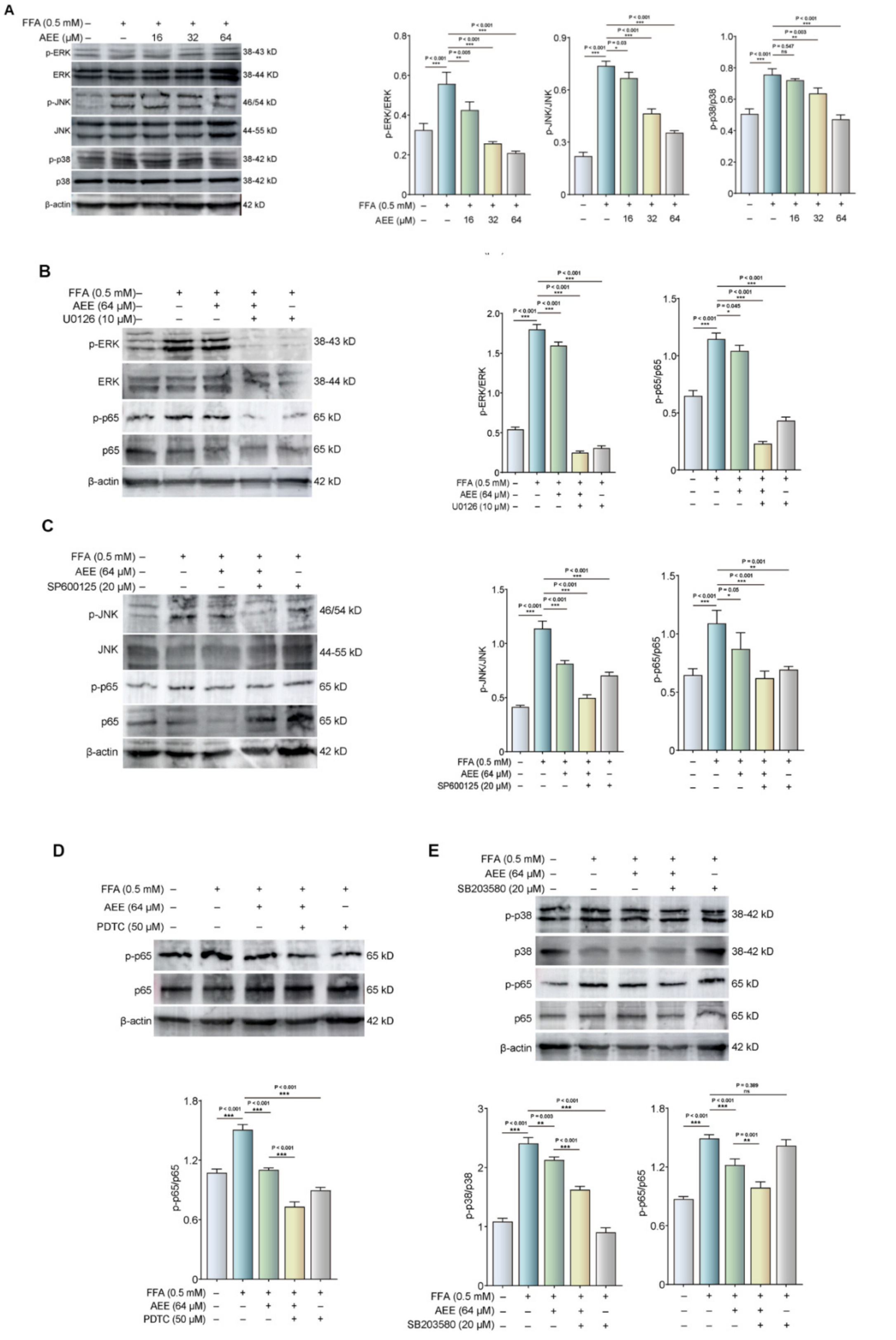

Supplementary Figure S2. Effects of U0126, SP600125, PDTC, and SB203580 on the phosphorylation level of p65. (A) The protein expression levels of ERK, p-ERK, JNK, p-JNK, p38, and p-p38 were detected by Western blot. (B) The protein expression levels of ERK, p-ERK, p65, and p-p65 were detected by Western blot. (C) The protein expression levels of JNK,

p-JNK, p65, and p-p65 were detected by Western blot. (D) The protein expression levels of p65 and p-p65 were detected by Western blot. (E) The protein expression levels of p38, p-p38, p65, and p-p65 were detected by Western blot. Data are presented as mean ± SEM. n = 3 per group, independent biological replicates. * $P < 0.05$, ** $P < 0.01$, and *** $P < 0.001$.

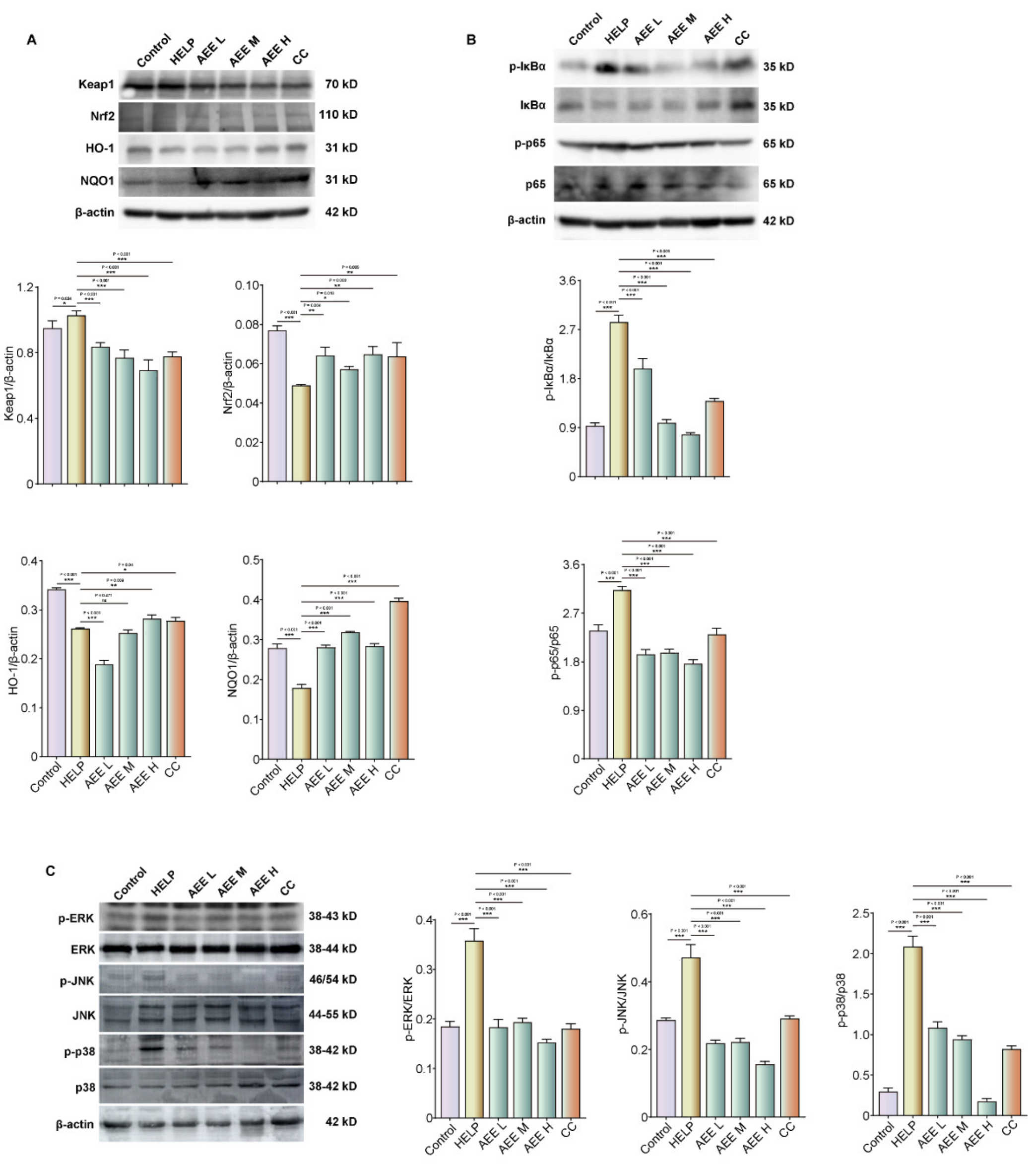

Supplementary Figure S3. Effects of AEE on Nrf2 and MAPK/NF-κB signaling pathways in HELP-induced laying hens. (A) The protein expression levels of Keap1, Nrf2, HO-1, and NQO1 were detected by Western blot. (B) The protein expression levels of p-IκBα, IκBα, p65, and p-p65 were detected by Western blot. (C) The protein expression levels of ERK, p-ERK, JNK, p-JNK, p38, and p-p38 were detected by Western blot. Data are presented as mean ± SEM. n = 3 per group, independent biological replicates. * $P < 0.05$, ** $P < 0.01$, and *** $P < 0.001$.

Supplementary Table S1 Antibodies used for western blot analysis (data from official website).

Antibody	Supplier	Catalogue number	Host species	Working dilution	Specificity	Application validation
β-actin	Proteintech	20536-1-AP	Rabbit	1:3000	Chicken	WB
Nrf2	Proteintech	16396-1-AP	Rabbit	1:1000	Chicken	WB
Keap1	Proteintech	10503-2-AP	Rabbit	1:1000	Chicken	WB
HO-1	Proteintech	10701-1-AP	Rabbit	1:1000	Chicken	WB
NQO1	Proteintech	11451-1-AP	Rabbit	1:1000	Chicken	WB
SOD1	Proteintech	10269-1-AP	Rabbit	1:1000	Chicken	WB
p65	Abmart	T55034S	Rabbit	1:1000	Chicken	WB
p-p65	Abmart	TP56372S	Rabbit	1:1000	Chicken	WB
IκBα	Proteintech	10268-1-AP	Rabbit	1:1000	Chicken	WB
p-IκBα	Proteintech	82349-1-RR	Rabbit	1:1000	Chicken	WB
COX-2	Proteintech	12375-1-AP	Rabbit	1:1000	Chicken	WB
IL-1β	Proteintech	26048-1-AP	Rabbit	1:1000	Chicken	WB
ERK	Proteintech	11257-1-AP	Rabbit	1:1000	Chicken	WB
p-ERK	Proteintech	28733-1-AP	Rabbit	1:1000	Chicken	WB
JNK	Abmart	T40073S	Rabbit	1:1000	Chicken	WB
p-JNK	Abmart	T40074S	Rabbit	1:1000	Chicken	WB
p38	Proteintech	14064-1-AP	Rabbit	1:1000	Chicken	WB
p-p38	Proteintech	28796-1-AP	Rabbit	1:1000	Chicken	WB
